# Parenteral treprostinil in paediatric pulmonary arterial hypertension: a systematic review and meta-analysis

**DOI:** 10.1183/16000617.0033-2025

**Published:** 2026-03-25

**Authors:** Julie Wacker, Raphael Joye, Maurice Beghetti

**Affiliations:** 1 Paediatric Cardiology Unit, Service of Pediatric Specialties, Department of Woman, Child and Adolescent Medicine, Geneva University Hospitals and Faculty of Medicine, University of Geneva, Geneva, Switzerland; 2 Pulmonary Hypertension Program, Geneva University Hospitals, Geneva, Switzerland

## Abstract

**Background:**

Paediatric pulmonary arterial hypertension (PAH) shares commonalities with adult disease but is essentially different regarding complexity and is usually more challenging to treat. Current treatment recommendations are based on expert opinion, small-scale paediatric studies and knowledge and consolidated guidelines for adults. Parenteral prostacyclins are recommended for high-risk patients but evidence is limited to cohort studies and retrospective data evaluations. The aim of this article was to summarise the available evidence on the efficacy and safety of parenteral treprostinil for paediatric PAH through a systematic review and to evaluate selected efficacy end-points through meta-analysis.

**Method:**

A systematic literature search (January 2000–April 2024) was conducted in PubMed, Google Scholar and clinical trial registries. Eligible studies included those reporting long-term outcomes of parenteral treprostinil in children with PAH. Moreover, a meta-analysis of selected efficacy end-points was performed based on published results from studies meeting predefined criteria.

**Results:**

32 studies encompassing 766 paediatric PAH patients treated with parenteral prostacyclins were identified; 649 patients received treprostinil. The meta-analysis was based on five publications including a total of 143 treprostinil-naïve patients. Despite the lack of randomised controlled trials, available data clearly indicate a treatment benefit of parenteral treprostinil in paediatric PAH. Literature data are supported by statistically significant results in the meta-analysis for PAH-relevant efficacy end-points.

**Conclusion:**

Based on currently available published data, parenteral treprostinil is effective and safe in the treatment of paediatric PAH.

## Shareable abstract

**Despite a long history of use of parenteral treprostinil in treatment of paediatric PAH, evidence is limited. This work summarises available efficacy and safety data, including a meta-analysis of efficacy end-points.** https://bit.ly/48EEBq8

## Introduction

Paediatric pulmonary hypertension (PH) shares common features with adult disease but is often associated with several comorbidities and exhibits a different spectrum of aetiologies to PH diagnosed in adulthood [1–3]. The reported annual incidence for paediatric PH is 64 per million children. Pulmonary arterial hypertension (PAH) is the most frequent type, but transient forms of PAH (*i.e.* persistent pulmonary hypertension of the newborn (PPHN) or repairable cardiac shunt defect) account for most of the reported cases. Of the remaining children with PAH, most have idiopathic PAH (IPAH), hereditary PAH (HPAH) or PAH associated with congenital heart disease (PAH-CHD). The reported incidences of IPAH/HPAH and non-transient PAH-CHD are 0.7 and 2.2 per million children, respectively, with a prevalence of 4.4 and 15.6 per million children [1].

Compared to adult PAH, paediatric PAH tends to show higher ratios of mean pulmonary arterial pressure (mPAP) to mean systemic blood pressure and pulmonary vascular resistance (PVR) to systemic vascular resistance at diagnosis, but then develops into right heart failure at later stages [4]. Historically, the prognosis for IPAH/HPAH appeared to be worse for children, with median survival estimated to be 10 months, compared to 2.8 years in adults [5, 6]. However, registries from the period prior to the availability of targeted PAH treatments showed comparable or even better survival estimates for paediatric patients than for adults in the same era [4].

The use of targeted PAH therapies has led to significant improvements in survival both in adults and children [5, 6], with reported survival rates for children of 96% at 1 year and 89% at 3 years [7]. These survival rates are notably higher than those reported for adults, of 87% at 1 year and 67% at 3 years [8]. More recent real-world data in paediatric PAH reported survival rates free from lung transplantation, atrial septostomy and Potts shunt intervention of 83.9%, 75.2% and 71.8% at 1, 3 and 5 years, respectively [9]. Although survival has considerably improved over the past decades, the rates remain unsatisfactory, highlighting the need for further treatment optimisation. The ultimate goal of treatment remains the improvement of both survival and quality of life.

In Europe, only oral medicinal products targeting the nitric oxide pathway or endothelin pathway, or acting as soluble guanylate cyclase stimulator are authorised for use in children. Prostacyclin medications, despite being recommended for more advanced forms of paediatric PAH, are not authorised. Findings from paediatric studies and observational cohort studies indicate that treatment algorithms commonly used in adults are also effective in children, including the superiority of combination therapy over monotherapy, resulting in the off-label use of PAH treatments in the paediatric population. This is especially true for parenteral prostacyclin analogues, which are currently recommended as an upfront combination treatment (with oral medications) for high-risk patients at baseline and as escalation treatment for intermediate-high/high-risk patients at follow-up [10, 11]. Epoprostenol has been the mainstay of therapy in advanced forms of PAH for many years. Treprostinil, with the advantages of a longer half-life and greater stability, and which is available as a subcutaneous or intravenous formulation, has been increasingly used with proven efficacy in adults. Treprostinil is also widely reported to be used in children as a chronic therapy for progressive forms of PAH, and as a short-term treatment for PPHN [12].

This systematic review aims to provide a detailed summary of the efficacy and safety data reported in published literature on the use of parenteral treprostinil for the chronic treatment of PAH in the paediatric population. Additionally, we conducted a Bayesian meta-analysis of selected efficacy parameters in treatment-naïve paediatric PAH patients receiving parenteral treprostinil.

## Material and methods

We conducted a systematic review and meta-analysis of the literature on the use of parenteral treprostinil for the treatment of paediatric PAH (Group 1 PH), with a specific focus on long-term parenteral treprostinil administration.

Of note, treprostinil is also used as short-term therapy for transient PH, such as PPHN or repairable cardiac defects, which represents a substantial proportion of paediatric PH cases. Because this short-term treatment concept differs fundamentally from the continuous long-term therapy required for progressive PAH, these cases were excluded from the review.

### Data sources and searches

AOP Health supported the identification of relevant literature and conducted a search in two electronic databases (PubMed and Google Scholar) using a combination of Medical Subject Headings (MeSH) terms, including “treprostinil”, “prostanoid”, “prostacyclin”, “paediatric”, “children”, “pulmonary hypertension” and “pulmonary arterial hypertension”. Paediatric clinical trials registered on the European Union Clinical Trials Register and ClinicalTrials.gov that are using treprostinil as the investigational drug were also included. The defined timeframe for the search was 1 January 2000 until 30 April 2024. No restrictions regarding study design or location were set. Articles written in any language other than English were automatically excluded.

### Literature selection

Each title and abstract was reviewed by two independent reviewers for relevance, reporting consistency and data availability. The following types of articles were excluded: 1) systematic reviews, 2) meta-analyses, 3) pharmacokinetic or dynamic studies without clinical data, 4) investigations of PH therapies other than parenteral treprostinil, 5) investigations of other administration forms of treprostinil, 6) mixed adult and paediatric cohorts not reporting data separately, 7) investigations lasting <3 months and 8) investigations in other PH groups or PPHN.

Pre-selected publications were then reviewed in detail. Information on study design, data collection timeframe, patient numbers and diagnoses, route of treprostinil administration, number of patients receiving treprostinil and patient age at treprostinil initiation were extracted when available. Reported efficacy results and safety information were summarised.

### Meta-analysis

The selected literature was further screened for interpretable information on change from baseline in 6-min walk distance (6MWD), PVR, mPAP, tricuspid annular plane systolic excursion (TAPSE) and World Health Organization functional classification (WHO-FC). A Bayesian meta-analysis on these variables was then performed. Only full-text publications qualified for the meta-analysis.

The following eligibility criteria were applied: 1) patients with any type of PAH; and 2) at least one efficacy parameter (*i.e.* 6MWD, PVR/PVR index (PVRi), mPAP, TAPSE or WHO-FC) evaluated, meaning that either individual patient data or summary statistics were available. To reduce a potential bias due to inclusion of different PH aetiologies, only publications supporting the diagnosis of PAH using haemodynamic variables were included, except for one publication clearly stating IPAH as the diagnosis.

A Bayesian approach provides natural weighting of studies based on sample size and the simple combination of evidence from reported studies in the selected literature. Here, this provided information on the probability that treprostinil offers a clinically significant benefit, as reflected in each efficacy end-point. The clinical significance of the change was defined as follows: an increase of at least 50 m in the 6MWD, a decrease of at least 3 WU or WU·m^2^ for PVR or PVRi, a decrease in mPAP of at least 5 mmHg and an increase in TAPSE of at least 2 mm. The cut-off value for the 6MWD was based on the mean clinically meaningful differences evaluated in three studies [13–15]. Owing to the lack of valid cut-off values for PVR/PVRi, mPAP and TAPSE, the set values were assumed to be of clinical relevance based on our clinical experience.

For the four continuous variables (6MWD, PVR/PVRi, mPAP and TAPSE), a meta-analytic-predictive (MAP) distribution for the change since baseline in means and standard deviations was obtained. WHO-FC available data were binarised into mild limitation (WHO-FC I and II) and severe limitation (WHO-FC III and IV).

To validate the primary results, a robustness check using a sceptical prior was performed on exceedance probabilities and 95% credibility intervals (95% CrI). In addition, a sensitivity analysis was performed by excluding specific datasets, namely the most/least favourable for treprostinil treatment effect. A description of the exact method can be found in the supplementary material.

## Results

### Eligible literature

A total of 7123 records were identified through database searching. The PubMed search yielded 253 records. After applying automatic filters to remove duplicates, non-English articles and non-human studies, 162 records remained for title and abstract screening. Of these, 32 original research articles met the eligibility criteria, underwent full-text review and were included in the literature synthesis on long-term treatment with parenteral treprostinil in paediatric PAH [16–47].

The selected publications mostly reported data collected retrospectively, with only six prospective studies [16, 17, 22, 23, 40, 44]. The majority of publications were available as full-text, with one poster [26] and two abstracts [27, 31]. Data provided in the selected literature were collected between 1992 and 2023. The majority of publications collected data between 2010 and 2023. Eight publications did not report the data collection timeframe in detail [27–29, 33, 37, 41, 45, 47].

Out of the 32 eligible articles, 16 publications reported data on efficacy parameters [17–21, 24, 25, 27, 29–31, 34, 36, 39, 42, 46]. Out of these 16 publications, five were included in the meta-analysis [17–19, 24, 25].

Of the 32 articles, 13 reported relevant information on the safety and tolerability of parenteral treprostinil in paediatric PAH patients [16, 18, 25, 26, 28–33, 38, 44, 45].

The literature search results are presented in detail in figure S1.

### Patient characteristics

Based on selected literature, the use of treprostinil in a paediatric population was first reported by Ivy *et al.* [30], who successfully switched 13 children with various PAH forms from intravenous epoprostenol to intravenous treprostinil.

The selected literature included a population of 766 paediatric patients with PAH treated with parenteral prostacyclins. Parenteral treprostinil was clearly reported to be administered in 649 patients and nine patients received inhaled treprostinil. In five publications, patients also received other prostacyclins without reporting data for treprostinil only [19, 20, 34, 42, 43].

**FIGURE 1:**
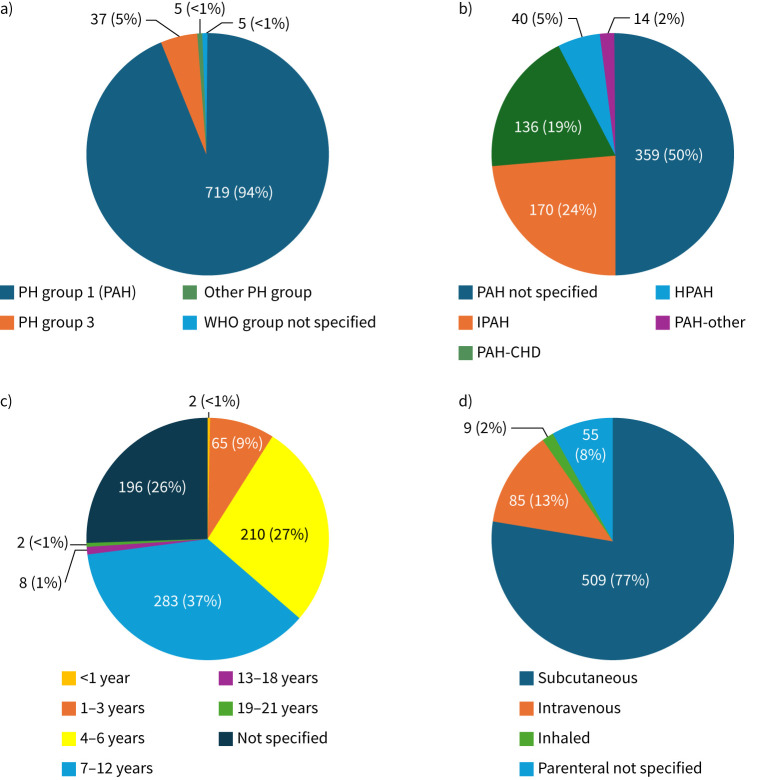
a) Reported pulmonary hypertension (PH) group diagnosis at baseline (n=766). b) Pulmonary arterial hypertension (PAH) subtype distribution (n=719). c) Age at treatment (n=766). Age stratification based on age groups used in the Pediatric Quality of Life Inventory (PedsQL version 4.0) questionnaire; cut-off age for pediatric population was 21 years. d) Distribution of patients on treprostinil per mode of administration (n=658). The remaining 108 of the total 766 patients did not receive parenteral treprostinil or prostacyclin medication was not specified. HPAH: hereditary pulmonary arterial hypertension; IPAH: idiopathic pulmonary arterial hypertension; PAH-CHD: pulmonary arterial hypertension associated with congenital heart disease; PAH-other: other associated pulmonary arterial hypertension; WHO: World Health Organization.

Even though other PH types were excluded from the review, several articles reported mixed populations, including various forms of PAH in some patients [16, 19, 25, 30, 36, 40, 42, 47]. From the overall population, 719 (94%) had a clear diagnosis of PAH. The subtype of PAH was not provided for half the population. The most common PAH subtype reported was IPAH (24%), and the preferred administration route was subcutaneous (77%), as detailed in figure 1. Other associated PAH was reported for a small proportion (2%) of patients, including two patients with pulmonary veno-occlusive disease [20], three with porto-pulmonary hypertension [29, 42], two with PAH associated with connective tissue disease [34] and seven with associated PAH but not further specified [43].

The reported age ranged from 0.1 to 21 years at treatment initiation. We categorised the patients into commonly used age groups, resulting in the highest percentage (37%) of patients in the age group 7–12 years, followed by 27% in the 4–6 years group; age at treatment initiation was not specified for 26% of patients (figure 1). Of note, one publication reported a median age of 2.6 years, ranging from 0.02 to 29 years, indicating that some patients older than 21 years have been included as well [19]. All publications listed in the selected literature intended long-term sustained treprostinil therapy, achieving median treatment periods ranging from 2.1 to 60 months. Reported doses were noted to be highly variable throughout the literature, ranging from 21 to 170 ng·kg^−1^·min^−1^. However, median doses usually did not exceed 100 ng·kg^−1^·min^−1^, except in a few publications [24, 32, 34, 42]. Higher doses were generally attributed to prolonged treatment duration and disease progression.

Parenteral treprostinil is indicated for high-risk patients at diagnosis and for treatment escalation in patients not achieving the treatment goal at follow-up [10]. Most patients received parenteral treprostinil as second-line or third-line therapy, except for in one publication where treprostinil was used as first-line monotherapy [24]. Different publications including 13 patients used upfront triple combination therapy constituting oral drugs and parenteral treprostinil [18, 22].

Detailed patient characteristics are in figure 1.

### Efficacy overview

Efficacy data have been reported for 359 patients [17–21, 24, 25, 27, 29–31, 34, 36, 39, 42, 46].

Change in WHO-FC was reported for 254 patients in 10 publications [17–21, 25, 27, 30, 34, 36]. WHO-FC improved or remained stable at the last follow-up in most patients. Notably, at baseline, 74% of the patients were classified in WHO-FC III–IV, whereas at the last follow-up, only 28% of patients were in this group. In three studies, improvement in WHO-FC was statistically significant [18–20].

Change in 6MWD was reported for 165 patients in six publications, with an improvement of up to 222 m [17–20, 30, 31]. In four studies, the improvement was statistically significant [17–20].

Changes in PVRi were reported for 212 patients in eight publications [18, 20, 24, 31, 34, 39, 42, 46], whereas PVR was reported for 27 patients in three publications [17, 21, 29]. Six studies reported a decrease in PVRi of >3 WU·m^2^ [18, 20, 24, 39, 42, 46], with three showing statistically significant improvements [18, 20, 42]. One study did not report mean or median values at follow-up but stated that PVRi dropped by ≥69% in 63% of patients [34]. One study evaluating patients pretreated with epoprostenol before their transition to intravenous treprostinil at baseline observed a PVRi increase, albeit not statistically significant [31]. Among the three studies assessing PVR, two reported reductions of >3 WU [17, 29], with one being statistically significant [17]. In one study, PVR remained stable [21].

Change in mPAP was evaluated for 252 patients in 12 publications [17, 18, 20, 21, 24, 29–31, 34, 39, 42, 46]. A decrease of >5 mmHg was shown in six studies [18, 20, 24, 29, 39, 42], of which four were clinically significant [18, 20, 24, 42]. One study reported improvement in mPAP by ≥25% in 53% of patients at follow-up without reporting mean or median values [34]. The remaining studies showed stable mPAP over the observation period except for two publications reporting an increase in mPAP at 12 months, both statistically nonsignificant [17, 31]. Additionally, Siehr *et al.* [24], who reported a clinically significant improvement from 58.3±19.7 mmHg to 41.1±17.7 mmHg at 24 months, observed an increase in mPAP at 48 months compared to baseline. This increase, however, was not statistically significant [24].

A detailed overview of the reported results is provided in tables 1 and 2.

**TABLE 1:** Overview of WHO-FC and 6MWD

Author	Patients, n BL/FU		FU, months		WHO-FC, n (%)									6MWD, m		
					BL				FU				p-value	BL	FU	p-value
	WHO-FC	6MWD	WHO-FC	6MWD	I	II	III	IV	I	II	III	IV				
**Ivy *et al*. [30]**	13/13	9/9	12	6	1 (8)	7 (54)	5 (38)	0	1 (8)	7 (54)	5 (38)	0	NR	516±115	513±10	NR
		9/8		12											565±84	NR
**Rowan *et al*. [31]**		10/6		12										452±59	448±73	NR
**Bajolle *et al*. [27]**	7/7		6		0	0	7 (78)	0	0	7 (78)	0	0	NR			
**Levy *et al*. [17]^#^**	10/10	7/7	4–36	12–24	0	1 (10)	4 (40)	5 (50)	2 (20)	6 (60)	1 (10)	1 (10)	NR	198.8±102.7	421.2±108.8	0.006
**Ablonczy *et al.* [25]**	8/8		11.4 (0.04–51.7)^+^		0	0	5 (63)	3 (37)	0	0	5 (63)	3 (37)	NR			
**Levy *et al*. [18]**	56/36	39/36	37	6	4 (7)		52 (93)		30 (83)		6 (17)		<0.0001	335±140	448±102	<0.0001
**Hopper *et al*. [19]^¶^**	30/34	11/11	12	1–3	16 (53)		14 (47)		27 (79)		7 (21)		0.047	457± 144.71	514.5±107.43	<0.001
		11/10		6–12											491.3±134.05	
**Haarman *et al*. [20]^¶^**	19/19	19/10	3	3	0	1 (5)	5 (26)	13 (68)	4 (21)	13 (68)	2 (11)	0	<0.001	262 (143–342)^+^	413 (372–488)^+^	<0.008
**Hollander *et al*. [21]**	16/16		8.3 (4.9–16.6)		0	4 (25)	6 (38)	6 (38)	0	12 (75)	3 (19)	1 (6)	NR			
**Tella *et al*. [34]^¶^**	30/29		38.3 (15.7–81.4)		7 (23)		23 (77)		17 (59)		12 (41)		NR			
**McSweeney *et al.* [36]**	40/38		19.1 (0.1–84)^+^		0	18 (45)	16 (40)	6 (15)	1 (3)	24 (63)	11 (30)	2 (5)	NR			

Follow-up time is presented as median or median (IQR), unless otherwise indicated. 6MWD data are presented as mean±sd, unless otherwise indicated. 6MWD: 6-min walk distance; BL: baseline; FU: follow-up; NR: not reported; WHO-FC: World Health Organization functional class. ^#^: total population n=60, but only 10 patients received treprostinil, results in the table are reported for treprostinil subset only. Authors did not perform a hypothesis test. From the reported individual patient data (n=10), we performed a paired t-test and obtained a statistically significant change from baseline in 6MWD (p=0.006, 95% CI 92.1–353.6). ^¶^: also included patients treated with epoprostenol providing results for the total population only. ^+^: reported as median (range).

**TABLE 2:** Overview of change in PVR/PVRI and mPAP

Author		Patients, n BL/FU		FU, months	PVRi, WU·m^2^/PVR, WU			mPAP, mmHg		
		PVR	mPAP		BL	FU	p-value	BL	FU	p-value
**Ivy *et al*. [30]**		–	13/6	12	–	–	–	52±13	49±13	NR
**Rowan *et al*. [31]**		16	16	3	10±7	9±5	ns	52±15	51±21	ns
				12		14±10			61±23	
**Levy *et al*. [17]^#,¶^**		7/7	8/8	12	21.4±7.7	15.9±7.7	0.04	63.3±12.3	68.8±14.9	ns
**Siehr *et al*. [24]**		20	20	12 (n=NR)	15.6±10.8	11.5±7.7	ns	58.3±19.7	49.7±17.1	ns
				24 (n=12)		7.6±3.7	ns		41.1±17.7	0.04
				48 (n=5)		12.9±0.8	ns		62.0±14.1	ns
**Levy *et al*. [18]^+^**		56/25	56/25	6	16±10	12±10	<0.001	63±20	50±28	<0.01
**Serrano *et al*. [29]^¶^**		1/1	1/1	2.1	16.75	2.38	NR	72	27	NR
**Haarman *et al*. [20]^§^**		10/10	10/10	6.8 (5.5–7.2)	18.6 (13.8–22.5)	9.2 (5.8–14.5)	<0.05	72 (62–99)	48 (40–70)	<0.05
**Hollander *et al*. [21]^¶^**		16/16	16/16	8.3 (4.9–16.5)	3.8 (3.1–5)	3.1 (2–4.4)	0.03	26 (17–38)	24 (17–27)	ns
**Tella *et al*. [34]^§^**		31/30	31/30	58.7±61.6 38.3 (15.7–81.4)	23.0±10.8 22.0 (14.0–29.2)	Reduction by ≥69% (19)	NR	71.2±20.4 71 (55–88)	Reduction by ≥25% (16)	NR
						Reduction by <69% (11)	NR		Reduction by <25% (15)	NR
**Sullivan *et al.* [46]**		6/6	6/6	8.1 (3.7−9.2)	5.0 (1.6–6.1)	1.7 (1.4–3.1)	ns	17 (14.8–18.8)	16 (13.5–21.0)	ns
**Miles *et al*. [42]^+^**		4/4	4/4	2.9 (2.1–4.4)	43.5 (19.5–67.0)	22.3 (16.5–32.5)	ns	72 (67–77)	64 (56–87)	ns
		14/14	14/14	4.3 (3.3–4.6)	13.7 (7.8–17.8)	6.7 (4.8–8.6)	0.003	56 (50–61)	49 (30–66)	0.002
**Kochanski *et al.* [39]^ƒ^**	**<6 years**	17/14	17/14	11.3 (0.8–40.9)^##^	15 (3.2–34.5)^##^	7.0 (1.7–19.0)^##^	0.005	61 (40–109)^##^	49 (16–95)^##^	ns
	**≥6 years**	23/18	23/18	12 (0.6–17.6)^##^	18 (6.3–43.0)^##^	12.8 (2.8–39.0)^##^	0.005	63 (30–102)^##^	61 (24–100)^##^	ns

Data are presented as mean±sd or median (interquartile range), unless otherwise indicated. BL: baseline; FU: follow-up; mPAP: mean pulmonary arterial pressure; NR: not reported; ns: nonsignificant; PVR: pulmonary vascular resistance; PVRi: pulmonary vascular resistance index. ^#^: total population n=60, but only 10 patients received treprostinil, results in the table are reported for treprostinil subset only. Authors did not perform a hypothesis test for PVR and mPAP but reported individual patient data. We applied a paired t-test and obtained a statistically significant mean PVR reduction from baseline by −5.8 WU (p=0.04, 95% CI −11.26– −0.31) and a mean mPAP increase from baseline of 5.5 mmHg, which was not statistically significant (p=0.153, 95% CI −2.62–13.62). ^¶^: reported PVR (WU). ^+^: included in total 22 patients, five patients without and 17 with supranormal cardiac index (>4 L·min^−1^·m^−2^) at BL; RHC was done for four and 14 patients, respectively. All patients received parenteral prostanoids without further specification of drug. ^§^: also included patients treated with epoprostenol, providing results for the total population only. ^ƒ^: population divided in age <6 years (n=17) and ≥6 years (n=23) at baseline. ^##^: reported as median (range).

### Safety overview

From the selected literature, 13 publications reported data on tolerability and safety, each noting at least one adverse drug reaction associated with treprostinil, comprising a total of 200 paediatric patients with PAH [16, 18, 25, 26, 28–33, 38, 44, 45]. Based on these publications, we summarised the reported frequencies of both systemic adverse drug reactions and reactions related to the mode of administration (*i.e.* subcutaneous or intravenous) (tables S3 and S4). To provide a rough indication for the safety profile in paediatric PAH patients, we compared the frequency to safety data reported in the adult population [48–56].

Systemic side-effects reported in 200 paediatric patients with PAH were in general expected and consistent with the safety events mentioned in treprostinil product characteristics. Interestingly, frequencies of gastrointestinal (diarrhoea, nausea, vomiting), musculoskeletal and connective tissue (joint, muscle, jaw pain), and nervous system (headache/dizziness, insomnia) disorders were remarkably lower in children than in adults (24% *versus* 70%, 15% *versus* 46% and 14% *versus* 31%, respectively). Frequencies of skin (rash, itching) and vascular (flushing, swelling/oedema) disorders were slightly higher than in adults (20% *versus* 9% and 29% *versus* 4%, respectively). Dose reduction followed by slow re-titration was reportedly a successful management strategy for systemic side-effects [16, 25, 29].

Beside systemic adverse reactions, treprostinil is known to cause administration route-specific events. Within the literature, subcutaneous-related events were evaluated for 152 cases and intravenous-related events were evaluated for 54 cases. Six patients were switched from subcutaneous to intravenous treprostinil, with safety events noted for each administration route. Subcutaneous administration of treprostinil is known to cause infusion site pain and other reactions, which were reported in 122 patients (80%) and five patients (3%) receiving subcutaneous treprostinil, respectively. These frequencies are lower compared to adult data: 93% for pain and 81% for other reactions. Local pain and other reactions were successfully managed with local or oral anaesthetics together with nonpharmacological strategies including cooling packages and change of infusion site [18, 25, 28]. Similar to adults, local pain and reactions were independent from treprostinil dose, flow volume and concentration [16, 18, 25]. It has been reported that the severity of infusion site pain decreases after the first 2–5 days following infusion site change [18], indicating a benefit of longer maintenance of the infusion site. Local site infections were reported in 13 paediatric patients (9%), compared to only 5% of the adult population. Antibiotic therapy was required in 12 cases, and surgical drainage in two [18].

In contrast to subcutaneous administration, the intravenous route is not associated with local pain but bears the risk of central line infections (CLIs) and bloodstream infections (BSIs). Such events were reported in 14 paediatric patients (26%) of the intravenous population, two of them being fatal. Importantly, 11 of the 14 cases were reported in the first two publications investigating the transition from intravenous epoprostenol to intravenous treprostinil [30, 31]. In adults, CLIs/BSIs have been reported in about 4% of cases [49–56].

A total of 26 patients (13%) were either switched to another mode of administration while continuing treprostinil therapy (six patients) or discontinued treprostinil therapy completely (20 patients) owing to side-effects. Out of 13 patients originally treated with subcutaneous treprostinil, six patients were switched to the intravenous route, whereas five were transitioned to intravenous epoprostenol and two patients were further treated with oral prostacyclin receptor agonists. Subcutaneous administration was mostly discontinued owing to infusion site pain, followed by local infections and reduced quality of life (QoL). Out of the 13 patients discontinuing intravenous treatment, two died due to CLIs. Three patients were switched to other intravenous prostacyclins, two changed therapy to inhaled iloprost and six patients were switched to oral prostacyclin receptor agonists. Patients were changed to inhaled or oral therapy mostly owing to reduced QoL.

Alternatively implantable pump systems can be used for intravenous treprostinil therapy, with their use reported in four adolescents [32, 33]. In three cases the patients were implanted due to local site effects attributed to subcutaneous therapy, whereas in one case the patient did not agree to any infusion *via* external pump systems at treatment start. Only minor post-surgery adverse events [33] and one catheter dislocation [32] were reported. For all four patients, implantable pumps were considered a safe option for treprostinil administration, leading to improved QoL [33] and improvements of PAH symptoms [32].

### Meta-analysis

Five studies met the eligibility criteria for the Bayesian meta-analysis of 6MWD, WHO-FC, PVR/PVRi, mPAP and TAPSE to evaluate the treatment effects of parenteral treprostinil in paediatric PAH (table 3) [17–19, 24, 25]. All five studies provided high-quality data for 143 treprostinil-naïve patients. Among these, 120 patients (84%) were confirmed to have PAH, with the predominant subgroup being IPAH (53%). At treprostinil initiation, patients were mainly in WHO-FC III and IV, reflecting the treatment algorithm of the international guidelines. There was heterogeneity in the time from diagnosis to treprostinil initiation. All publications included only patients <18 years old at the start of treatment, with the exception of one, which reported an age range of 0.02 to 29 years [19]. We summarised the results of the meta-analysis in table 4, including weighted means and sds for 6MWD, PVR/PVRi and mPAP (treated as continuous variables) and point estimates for WHO-FC (treated as a categorical variable). The results of TAPSE are provided in the supplementary material.

**TABLE 3:** Literature overview considered for the meta-analysis

Author	Design	Reported end-points	Paediatric population	Total, n	Route of application	Follow-up	Age, years
**Ablonczy *et al*. [25]**	R	WHO-FC	PH Group 1: 7 IPAH PH Group 3: 1 DLD^#^	8	*s.c.*	1 day–4.3 years	Median 12.4, range 2.6–14.7
**Hopper *et al*. [19]**	R	6MWD WHO-FC TAPSE	PH Group 1: 17 IPAH 1 HPAH 9 PAH-CHD PH Group 3: 21 DLD PH Group 4: 1 CTEPH	49^¶^	33 *s.c.*/*i.v.* 9 INH	1–3 months, 6–12 months	Median 2.6, range 0.02–29
**Levy *et al*. [17]**	P, OL	6MWD WHO-FC PVR mPAP	PH Group 1: 6 IPAH 4 PAH-CHD	10	*s.c.*	6MWD 4–36 months PVR within 1 year	Median 5.5, range 1.2–13
**Levy *et al*. [18]**	R	6MWD WHO-FC PVRi mPAP TAPSE	PH Group 1: 23 IPAH 13 HPAH 20 PAH-CHD	56	*s.c.*	6 months	Mean±sd 8±5.6 median 7
**Siehr *et al*. [24]**	R	PVRi mPAP	PH Group 1: 11 IPAH 9 PAH-CHD	20	*s.c.*	1 year, 2 years	Mean±sd 9.7±5.8

6MWD: 6-min walk distance; CTEPH: chronic thromboembolic pulmonary hypertension; DLD: developmental lung disease (consisting of congenital diaphragmatic hernia or bronchopulmonary dysplasia); HPAH: hereditary pulmonary arterial hypertension; INH: inhaled; IPAH: idiopathic pulmonary arterial hypertension; *i.v.*: intravenous; mPAP: mean pulmonary artery pressure; OL: open label; P: prospective; PAH-CHD: pulmonary arterial hypertension associated with congenital heart disease; PH: pulmonary hypertension; PVR: pulmonary vascular resistance; PVRi: pulmonary vascular resistance index; R: retrospective analysis; *s.c.:* subcutaneous; TAPSE: tricuspid annular plane systolic excursion; WHO-FC: World Health Organization Functional Class. ^#^: diagnosed with PAH associated with pulmonary hypoplasia and complex congenital malformations; ^¶^: 7 out of 49 patients (14%) were treated with *i.v.* epoprostenol, no subgroup analysis or individual patient data provided.

**TABLE 4:** Results of meta-analysis for 6MWD, PVRi/PVR, mPAP and WHO-FC efficacy end-points

End-point	Type of analysis	Selected publications	Prior^#^	Statistical method	MAP, mean±sd	95% CrI	Exceedance probability^¶^	p-value^+^	95% CI I^2^, %^§^
**6MWD**	Primary	Levy [17], Levy [18], Hopper [19] 6–12 m	Flat	Sample size	112.5±23.3	66.9–158.1	0.996		
				Precision	120.0±23.3	74.3–165.6	0.999	<0.001	(0.0–89.4)
	Worst case	w/o Levy [18]	Flat	Sample size	111.4±41.4	30.3–192.6	0.931		
				Precision	135.0±41.4	53.9–216.2	0.980	0.0011	(17.7–95.5)
	Best case	w/o Hopper [19] 6–12 m	Flat	Sample size	130.9±25.2	81.5–180.2	0.999		
				Precision	134.8±25.2	85.4–184.2	0.9996	<0.001	(0.0–92.5)
	Inclusion bias	with Hopper [19] 1–3 m instead of 6–12 m	Flat	Sample size	115.8±22.9	71.0–160.6	0.998		
				Precision	120.98±22.9	76.2–165.8	0.999	<0.001	(0.0–87.9)
	Prior robustness	Levy [17], Levy [18], Hopper [19] 6–12 m	Sceptical	Sample size	102.3±22.2	58.8–145.8	0.991		
				Precision	109.1±22.2	56.6–143.6	0.996		
**PVRi/PVR**	Primary	Levy [17], Levy [18]^ƒ^, Siehr [24] 1 year	Flat	Sample size	−4.25±1.65	−7.49– −1.02	0.776		
				Precision	−4.32±1.65	−7.56– −1.08	0.788	0.0089	(0.0–89.6)
	Worst case	w/o Levy [18]^ƒ^	Flat	Sample size	−4.54±2.41	−9.26–0.18	0.739		
				Precision	−4.68±2.41	−9.397–0.035	0.758	0.052	n.c.
	Best case	w/o Siehr [24] at 1 year	Flat	Sample size	−4.33±1.99	−8.23– −0.44	0.749		
				Precision	−4.42±1.99	−8.31– −0.53	0.763	0.026	n.c.
	Inclusion bias	with Siehr [24] at 2 years instead of 1 year	Flat	Sample size	−5.60±1.57	−8.67– −2.52	0.951		
				Precision	−5.77±1.57	−8.84– −2.697	0.961	0.00023	(0.0–89.6)
	Prior robustness	Levy [17], Levy [18]^ƒ^, Siehr [24] 1 year	Sceptical	Sample size	−3.86±1.57	−6.94– −0.78	0.708		
				Precision	−3.93±1.57	−7.01– −0.85	0.723		
**mPAP**	Primary	Levy [17], Levy [18], Siehr [24] 1 year	Flat	Sample size	−8.80±3.52	−15.70– −1.89	0.860		
				Precision	−6.29±3.52	−13.19–0.61	0.643	0.074	(0.0–87.1)
	Worst case	w/o Levy [18]	Flat	Sample size	−4.14±4.46	−12.88–4.59	0.424		
				Precision	−2.25±4.46	−10.99–6.45	0.269	0.61	(0.0–89.2)
	Best case	w/o Levy [17]	Flat	Sample size	−11.04±4.11	−19.09– −2.98	0.929		
				Precision	−10.56±4.11	−18.62– −2.51	0.912		n.c.
	Inclusion bias	with Siehr [24] 2 years instead of 1 year	Flat	Sample size	−11.71±3.54	−18.65– −4.77	0.971		
				Precision	−9.296±3.54	−16.24– −2.36	0.888		(0.0–91.2)
	Prior robustness	Levy [17], Levy [18], Siehr [24] 1 year	Sceptical	Sample size	−8.00±3.35	−14.59– −1.41	0.814		
				Precision	−5.72±3.35	−12.29–0.85	0.585		

End-point	Type of analysis	Selected publications	Prior^#^	Statistical method	Point estimate	95% CrI	Exceedance probability (>0.4)^¶^	Exceedance probability (>0.5)^¶^	p-value^+^	95% CI I^2^, %^§^
**WHO-FC**	Primary	Levy [17], Levy [18], Ablonczy [25], Hopper [19] 6–12 m	Flat β (1, 1)	Bayesian	0.542	0.425–0.647	0.993	0.775		
				Frequentist	0.531	0.439–0.622			<0.001	(86.0–96.8)
	Worst case	w/o Levy [18]	Flat β (1, 1)	Bayesian	0.322	0.136–0.494	0.191	0.021		
				Frequentist	0.260	0.124–0.395			<0.001	(65.2–95.6)
	Best case	w/o Ablonczy [25]	Flat β (1, 1)	Bayesian	0.588	0.470–0.695	0.999	0.932		
				Frequentist	0.654	0.552–0.756			<0.001	(61.4–95.3)
	Selection bias	With Hopper [19] 1–3 instead of 6–12 months	Flat β (1, 1)	Bayesian	0.503	0.380–0.616	0.948	0.522		
				Frequentist	0.523	0.429–0.616			<0.001	(87.4–97.0)
	Prior robustness	Levy [17], Levy [18], Ablonczy [25], Hopper [19] 6–12 months	Sceptical β (6, 6)	Robust Bayesian	0.495	0.378–0.601	0.945	0.467		
			Jeffreys β (0.5, 0.5)	Robust Bayesian	0.553	0.431–0.662	0.993	0.790		

6MWD: 6-min walking distance; CrI: credible interval; MAP: meta-analytic predictive; mPAP: mean pulmonary arterial pressure; PVRi: pulmonary vascular resistance index; PVR: pulmonary vascular resistance; WHO-FC: World Health Organization Functional Class. ^#^: initial priors for primary, the worst, best case and inclusion bias sensitivity analysis are flat, meaning that only the literature studies contribute any information to the MAP estimate. Sceptical initial prior was added for prior robustness check. The sceptical prior adds the same information as an imaginary extra study having precision equal to one out of 10 of the total precision in all our remaining studies, and mean 0 (*i.e.* the treatment had no effect) and zero-mean weighted by a sample size. ^¶^: probability of clinically significant improvement on treprostinil treatment. Clinical significance was defined as follows: for 6MWD an increase of ≥50 m, for PVR decrease of ≥3 WU, for mPAP a decrease of ≥5 mmHg, for WHO-FC a move to a less severe, *i.e.* WHO-FC I–II group, with a difference in proportions (mild *versus* severe) exceeding 40% and 50%, respectively. ^+^: a noninformative Bayesian (*i.e.* likelihood-based) solution was used for calculation of p-values of PVR, mPAP, common effect model for p-values of 6MWD end-point. ^§^: n.c. indicates that the confidence interval of I^2^ is not calculable, because the Cochrane Q statistic is either greater than number of studies k and the number of studies k<2 OR Cohrane Q ≤k and k≤2 (see also R library meta). ^ƒ^: Levy *et al.* [18] reported PVR in WU·m^2^, but individual patient data and body surface area (BSA) conversion factors were missing. Therefore, due in part to the comparable scales in the two datasets by the same first author, it was assumed that this BSA conversion factor can be thought of as roughly equal to 1.

#### 6MWD meta-analysis

The probability of treprostinil being associated with a clinically significant increase (≥50 m) in 6MWD was 99% based on the primary analysis. The 95% CrI of all sensitivity analyses, regardless of the choice of point estimate, remained above 0 and had at least 93% exceedance probability compared to the 50 m limit. Moreover, use of a sceptical prior in the precision-weighted method reducing the MAP mean by ∼10 m showed equal confidence intervals compared to the primary analysis with an exceedance probability >99%.

#### PVR/PVRi meta-analysis

The primary analysis of PVR or PVRi indicated a 78% probability that treprostinil provides a clinically significant improvement of at least 3 WU or 3 WU·m^2^ in the treatment of paediatric PAH. The probability of clinical benefit greatly increased from 78% to 95% after exchange of 1-year follow-up data to 2-year data reported by Siehr *et al.* [24]. Further sensitivity analysis confirmed the primary analysis results. However, exclusion of data reported by Levy *et al.* [18] reduced the point estimate by 0.29 WU but widened the 95% CrI.

#### mPAP meta-analysis

The data used for the primary analysis demonstrated a probability of 86% that treprostinil improves mPAP by 5 mmHg. However, the precision-weighted method did not yield a statistically significant exceedance probability despite indicating a positive effect of treprostinil. In line with the results obtained for PVR, the probability of mPAP improvement increased to 97% when the 2-year follow-up data reported by Siehr *et al.* [24] were used, leading to a p-value <0.05. By contrast, exclusion of data reported by Levy *et al.* [18] dropped the exceedance probability to 26% when applying the precision-weighted method, although not statistically significant.

#### WHO-FC meta-analysis

We calculated the probability of patients improving from WHO-FC III–IV to I–II. The probability of being in a less severe WHO-FC category (I–II) improved by 55% with treprostinil therapy, which was statistically significant. Several sensitivity analyses were performed, all of which were statistically significant and supported the primary analysis.

## Discussion

In general, our review was able to identify a substantial population of 766 paediatric patients with PAH who were treated long-term with parenteral prostacyclins. However, treprostinil treatment could be clearly allocated to only 86% of patients given that some publications reported overall data for mixed cohorts, including patients on other parenteral prostanoids or non-parenteral treprostinil [19, 20, 34, 43], or did not specify subgroup samples sizes for treprostinil and other prostanoids [42]. However, patients receiving treprostinil represented the largest proportions of these mixed cohorts. One publication reported nine patients on inhaled treprostinil, accounting for 1.4% of patients receiving treprostinil [19]. This relatively large number of patients indicates frequent use of parenteral treprostinil in paediatric PAH, with predominantly subcutaneous administration. Despite some inconsistency in reporting the diagnosis and age at treatment initiation, the majority of patients (94%) had a clear diagnosis of PAH and started treprostinil at a paediatric age, with the exception of one publication that included a few patients older than 21 years [19]. Treatment initiation before 1 year was reported for <1% of patients, confirming that progressive PAH is generally diagnosed in patients older than 1 year.

Most patients were categorised in WHO-FC III–IV, most probably stratified to high risk or intermediate-high risk. However, patients received upfront triple therapy in only two publications [18, 22], with oral phosphodiesterase-5 inhibitor (PDE5i) and endothelin receptor antagonist (ERA) as well as parenteral treprostinil, which is the currently recommended treatment strategy for all not low-risk adult patients with PAH [11]. This indicates that children are treated more cautiously with a stepwise approach no longer recommended in adult PAH. Future investigations are needed to confirm the tolerability and efficacy of upfront combination therapy in paediatric PAH.

This systematic review was inherently limited by the rarity of paediatric PAH, which restricts the availability of large, high-quality studies. The low prevalence of the condition results in small sample sizes, heterogeneous study designs, no randomised trials data available and variability in outcome reporting, all of which pose challenges to comprehensive synthesis of efficacy and safety. Furthermore, limited long-term observations, with inconsistent or no information on time of diagnosis, disease progression and treatment sequences and duration, precluded reliable survival analysis.

### Efficacy

Despite heterogenous treatment patterns, timing of initiation and dosing, parenteral treprostinil therapy showed improvement of clinically relevant efficacy parameters, *i.e.* 6MWD, WHO-FC, PVRi/PVR and mPAP. Although the heterogeneity of sample sizes (range 1–56) and follow-up assessment times (median range 2.1–48 months) across the studies and case reports must be acknowledged, 81% of publications accounting for 90% of investigated patients reported improvement in efficacy parameters [17–21, 24, 27, 29, 34, 36, 39, 42, 46]. In general, improvements were comparable or superior (6MWD) to results seen in adults [57].

Only three studies reported no improvement or worsening in efficacy parameters. Of those, one showed stabilisation of patients in end-stage disease [25]. The other two investigated the change from intravenous epoprostenol to intravenous treprostinil and showed safe transition and sustained stabilisation of the patients [30, 31]. However, the long previous treatment time with epoprostenol prior to transition (median 2.6 years, range 1.2–9 years) and thus long time since diagnosis might have contributed to a lack of improvement considering the progressive nature of PAH [30]. In the early trials reporting transition from intravenous epoprostenol to intravenous treprostinil, one could speculate that lack of experience with treprostinil and therefore potential underdosing may have played a role regarding worsening or non-improvement. However, this review did not aim to evaluate dose-response in efficacy parameters given the incomplete information on dosing in a substantial number of publications.

The key limitation of the literature used for the efficacy overview was the low proportion of prospectively collected data and absence of randomised controlled trials (RCTs). Given the predominantly retrospective character of published outcomes, a potential tendency to primarily report therapy success over failure cannot be excluded.

### Reported safety data

Throughout the literature, the use of treprostinil was reported to be safe and well tolerated. The observed systemic drug reactions were typical for prostaglandins in general and in line with the safety profile stated in the treprostinil product characteristics. Systemic side-effects were successfully managed by the well-established strategy of slight dose reduction until resolution and subsequent slow up-titration to the maximum tolerated dose.

As in adults, subcutaneous administration was much more common than intravenous treprostinil use (77% *versus* 13%), potentially due to the lower risk of infections or easier pump handling. Subcutaneous site pain and other reactions had a lower incidence in the paediatric population than in adults. Local infections, however, seemed to be more frequent, potentially because children may have difficulties adhering to hygiene requirements while engaged in typical activities (*e.g.* playing outdoors). Nevertheless, these local side-effects were resolved without treatment discontinuation in the vast majority of patients (95.4%). Moreover, local reactions were found to be dose independent [16, 18, 25], consistent with findings in adult populations [58]. There may be a benefit of longer site maintenance to improve tolerability because the pain seems to diminish after 2–5 days [18], and frequent site change seemed to be an intolerability factor for the majority of the patients who discontinued subcutaneous treprostinil [36].

BSIs and CLIs remain the most concerning drug reactions related to intravenous treprostinil administration. The relatively high incidence of CLIs (26%) reported in the paediatric PAH population treated with intravenous treprostinil might be explained by the fact that the majority of events occurred in the first reports on transitioning from intravenous epoprostenol, for which there was a lack of experience with treprostinil [30, 31]. For example, in adults, the initial high rate of BSIs related to intravenous treprostinil dropped to a similar rate as observed with intravenous epoprostenol following the introduction of closed-hub systems with protected connections [59] and a switch to a diluent with a basic pH [60]. If we were to exclude patients from these early reports, there would be a significant reduction in the frequency of CLIs and BSIs to 12%, which is still higher than the frequency observed in adults. However, the main reason to discontinue intravenous treprostinil therapy was limited QoL, and not CLI/BSIs, despite their high frequency, showing that these events, although serious, are manageable. Appropriate training of patients and caregivers in sterile preparation of the medication, operation of the pump and care of the central venous catheter is essential before starting intravenous treprostinil. An interesting aspect regarding CLI/BSI management is that treprostinil-associated infections were prevalently induced by gram-negative pathogens, which should be considered in treatment decisions [61]. Detailed standardised protocols for intravenous pump management would help minimise CLI/BSIs. Moreover, the use of implantable pumps might be an alternative option, reducing the risk for CLI/BSIs or local subcutaneous-related reactions while improving QoL, although limited for adolescent patients because of the size of the currently available pump systems.

However, we acknowledge limitations of our comparison of drug reaction incidence between paediatric and adult populations. We only included articles reporting at least one drug reaction in our safety evaluation, leading automatically to higher frequencies. We also had a reduced sample size of 200 paediatric patients. This is different from adult data that were collected prospectively with longer follow-up times in larger sample sizes.

### Meta-analysis

The results of the meta-analysis proved a significant treatment benefit of parenteral treprostinil in paediatric PAH and confirmed the trend observed in the literature regarding efficacy. The positive treatment effect of treprostinil was supported by the different sensitivity analyses.

Although the meta-analysis targeted paediatric patients, one study included a small proportion of individuals over 18 years old, with a reported median age of 2.6 years (range 0.02–29 years) [19], potentially constituting a bias for interpretation of paediatric data. However, considering this very low number of adult patients, we assumed negligible influence on quantitative meta-analysis.

To avoid bias of mixed cohorts consisting of patients treated with other prostacyclins, we aimed to include in the meta-analysis only publications investigating solely the efficacy of treprostinil treatment. The only exception was Hopper *et al.* [19], who also included patients treated with epoprostenol, but because these patients only accounted for 14% of the total study population it was deemed acceptable. All selected publications investigated parenteral prostacyclin-naïve patients except for Ablonczy *et al*. [25], who included two patients (25%) pretreated with intravenous iloprost for a short time (13 and 43 days, respectively); thus, pretreatment effect was considered negligible.

The cut-off values for improvement in PVR/PVRi (3 WU/3 WU·m^2^) and mPAP (5 mmHg) were chosen based on our clinical expertise. Even though these cut-off values might be questioned, a clear positive impact of treprostinil on these efficacy parameters was supported by reductions in point estimates greater than the cut-off values in primary and all sensitivity analyses except one, the worst-case scenario for mPAP.

The meta-analysis was performed without individual patient data because only two articles provided patient-level data [17, 25]. Even though no before-and-after correlation on a patient level could be derived from published summary statistics, it should be considered that, in general, this correlation would cause a reduction of the standard errors, resulting in even more significant findings. Therefore, the results of the presented meta-analysis can be thought of as lower bounds on the efficacy of treprostinil in the paediatric population with PAH.

## Conclusion

Efficacy and safety of parenteral treprostinil were reported in a high number of paediatric patients with PAH, showing consistent clinical improvement and good tolerability. Available data on the efficacy of treprostinil in paediatric PAH consist mainly of retrospective and small-scale studies. Our Bayesian meta-analysis was nevertheless able to demonstrate a positive treatment effect of parenteral treprostinil on 6MWD, WHO-FC and PVR/PVRi. More prospective trials with clearly defined eligibility criteria, homogenous populations, relevant sample sizes and long-term outcome reporting are warranted for future investigations of treprostinil efficacy and its impact on survival in paediatric PAH.

Available literature shows that treprostinil is well tolerated in children, with possibly less infusion site pain than in adults for subcutaneous delivery, and possibly more bloodstream infections in intravenous delivery. More data originating from prospective studies would be valuable to assess the safety profile.

Prospective evaluation of upfront *versus* add-on initiation of treprostinil on efficacy and safety are equally important to enhance management of PAH in vulnerable paediatric populations.

Although parenteral prostacyclin analogue use is supported by the international guidelines for severe PAH in children, the lack of robust evidence has hampered any marketing authorisation. The recently initiated prospective TREPaed trial (NCT06350032) investigating tolerability and safety of a paediatric treprostinil formulation may help fill this gap.

**Points for clinical practice and questions for future research**

- Parenteral treprostinil is an effective and safe treatment for progressive PAH in the paediatric population, particularly in high-risk patients.
- Children are treated more cautiously with a stepwise approach no longer recommended in adult PAH, because only a small proportion receive upfront triple therapy with oral PDE5i and ERA.
- Clinical benefits such as improved 6MWD, PVR, mPAP and WHO-FC reported in registries and retrospective studies support its use in paediatric PAH despite the lack of RCTs and no authorisation status.
- In home care, subcutaneous administration may be preferred because of the lower infection risks compared to intravenous therapy.
- Infusion site pain and reactions remain the main challenges associated with subcutaneous administration; however, they are reported less frequently in children than in adults.
- Infusion site pain and reactions can be managed effectively with local or oral anaesthetics, cooling packs and site changes. Pain decreases after 2–5 days, suggesting a benefit of longer site maintenance.
- Local pain and reactions are dose independent, consistent with findings in adult populations.
- CLIs/BSIs are major risks associated with intravenous prostacyclin treatment. Proper catheter care, sterile handling and standardised intravenous pump protocols are essential for prevention.
- Implantable pumps may be an alternative option in older children, reducing CLI/BSI risks from external intravenous pumps and local reactions from subcutaneous administration, while improving QoL, though more documented cases are needed.
- Further research is required to provide more robust evidence on the tolerability and efficacy of upfront combination therapies with prostacyclins specifically in paediatric PAH and to refine treatment initiation timing and dosing strategies.
- Given the lack of RCTs but extensive registry and real-world evidence, future treatment guidelines should explore leveraging existing data. However, more data from RCTs are warranted for future investigations to enable regulatory approval of paediatric-specific formulations.

## Supplementary material

**Please note:** supplementary material is not edited by the Editorial Office, and is uploaded as it has been supplied by the author. Supplementary material ERR-0033-2025.SUPPLEMENT
