# Supplementary material for: Parenteral treprostinil in paediatric pulmonary arterial hypertension: a systematic review and meta-analysis
Source: Eur Respir Rev. 2026 Mar 25;35(179):250033. doi: 10.1183/16000617.0033-2025 (PMC13014287; doi:10.1183/16000617.0033-2025)
Supplement: Supplementary file 1 [file ERR-0033-2025.SUPPLEMENT.pdf]

Supplementary Material

Parenteral Treprostinil in paediatric pulmonary arterial hypertension: a clinical review

Contents

1. Literature search ... 3
   1.1. Literature search flow ... 4
   1.2. Selected literature list ... 4
2. Statistical method for Bayesian meta-analysis ... 7
   2.1. Continuous variable analysis ... 7
   2.1.1. Prior robustness check ... 9
   2.2. Discrete variable analysis ... 10
   2.2.1. Dataset inclusion sensitivity analysis ... 11
3. Results ... 12
   3.1. Meta-analysis ... 12
   3.1.1. TAPSE ... 12
   3.2. Safety Overview ... 13
   3.2.1. Systemic adverse drug reactions ... 13
   3.2.2. Adverse drug reactions related to application route ... 15
4. References ... 16

1. Literature search

We performed the systematic literature review in the timeframe 1st January 2000 until 30th April, 2024 in 2 electronic databased (PubMed and Google Scholar) using a combination of the terms "treprostinil", "prostanoid", "prostacyclin", "paediatric", "children", "pulmonary hypertension" and "pulmonary arterial hypertension". The review was done in the steps summarized in **Figure S1** together with yielded number of publications after each review step. In addition, literature search resulted in 32 publications selected for the full text review. The publications and their methodological and reporting quality (text format, study design, period of data collection and relevance for the systemic efficacy and safety overview) is listed in **TABLE S1** Each of these publications contained relevant patient characteristics data.

1.1. Literature search flow

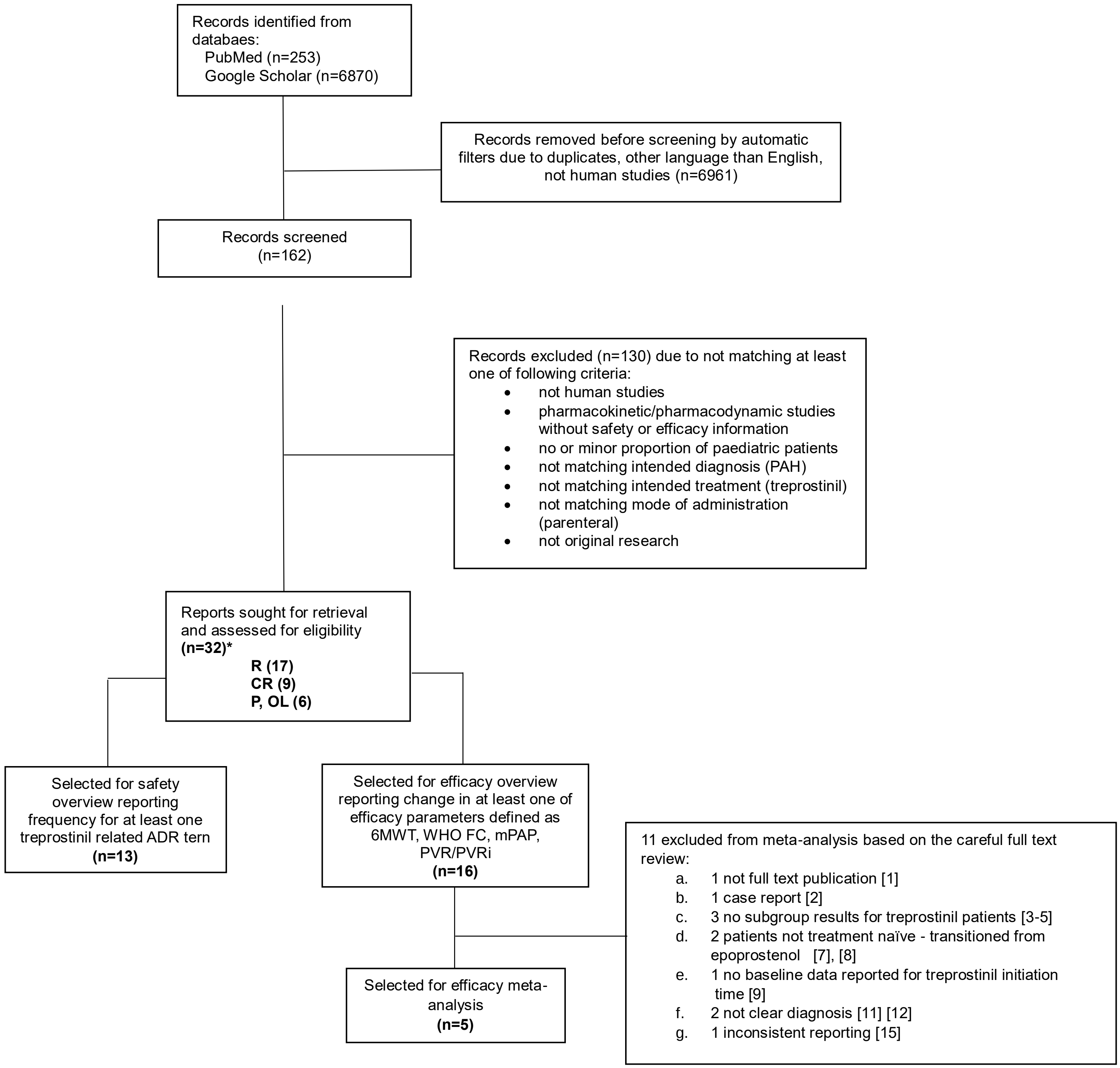

FIGURE S1: Flowchart for literature retrieval and the process of selection

* Due to specific key word combination and restricted number of published literature for use of treprostinil in paediatric PAH population, from all 32 records sought for retrieval, none was excluded. Data in all 32 publications were assessed for eligibility in full text review and considered as supporting data (diagnosis at baseline, age, treprostinil/prostanoid mode of administration). Although majority of publications were available as full text articles, one poster [6] and two abstracts [1, 8] are included in this selection.

R= retrospective, observational cohort studies, CR = case report, P, OL = prospective open-label studies, PAH= pulmonary arterial hypertension, ADR adverse drug reaction (related to treprostinil), 6MWT=6 minutes walking distance, WHO FC = World Health Organisation functional class, PVR= pulmonary vascular resistance, PVRi = pulmonary vascular resistance index, mPAP=mean pulmonary arterial pressure

1.2. Selected literature list

All publications listed below were considered for patient characteristics data (diagnosis, age, mode of administration, number of treated patients).

TABLE S1: Overview of selected literature (n=32)

Author	Publication Year	Format	Study conduct	Design	Relevant data
Ivy *et al.* [7]	2007	Full text	08.2004 – 08.2006	retro	EO, SO
Rowan *et al.* [8]	2008	Abstract	nk.2004-nk.2007	retro	EO, SO
Bajolle *et al.* [1]	2010	Abstract	nk	retro	EO
Smadja *et al.* [10]	2011	Full text	02.2008 - 02.2010	prosp, OL	
Levy *et al.* [13]	2013	Full text	02.2008 - 12.2010	prosp, OL	EO, MA
Siehr *et al.* [14]	2013	Full text	nk.1992 – nk.2010	retro	EO, MA
Vernetta *et al.* [6]	2017	Poster	nk.2011-nk.2017	retro	SO
Levy *et al.* [16]	2018	Full text	nk.2009-nk.2016	retro	EO, MA, SO
Hopper *et al.* [17]	2018	Full text	01.2001 - 08.2005	retro	EO, MA
Ablonczy *et al.* [18]	2018	Full text	nk.2006 – nk.2016	retro	EO, MA, SO
Hall *et al.* [19]	2019	Full text	11.2013 - 04.2016	prosp, OL	SO
Bacha *et al.* [20]	2019	Full text	02.2008 - 12.2010	prosp, OL	
Serrano *et al.* [2]	2019	Full text	nk	CR	EO, SO
Desole *et al.* [21]	2019	Full text	07.2012 – nk.2017	CR	SO
Hollander *et al.* [11]	2020	Full text	01.2012 - 04.2019	retro	EO
Ono *et al.* [22]	2020	Full text	nk	CR	SO
Álvarez-Fuente *et al.* * [23]	2020	Full text	nk	CR	SO
Tella *et al.* [3]	2020	Full text	04.1999 -04.2019	retro	EO
Rothman *et al.*** [24]	2020	Full text	nk	CR	
Haarman *et al.* [25]	2021	Full text	nk.2010-nk.2019	retro	EO
Jackson *et al.* [26]	2021	Full text	nk.2019	retro (nurse survey)	
Colglazier *et al.* *** [27]	2021	Full text	08.2016-03.2019	CR	SO
Douwes *et al.* [28]	2021	Full text	nk.2000-nk.2010	retro	
Handler *et al.* [29]	2022	Full text	nk.2015-nk.2019	prosp, OL	
Sullivan *et al.***** [12]	2023	Full text	03.2014-08.2021	retro	EO

Author	Publication Year	Format	Study conduct	Design	Relevant data
Domingo *et al.* ***** [30]	2022	Full text	nk	CR	
McSweeney *et al.* [9]	2023	Full text	01.2009-12.2019	retro	EO
Kavgaci *et al.* * [31]	2023	Full text	nk	CR	
Miles *et al.* [5]	2023	Full text	07.2005-07.2020	retro	EO
Nelson *et al.* [32]	2023	Full text	12.2021-01.2022	prosp, OL (survey caregivers)	SO
Faircloth *et al.* [33]	2023	Full text	nk	CR	SO
Kochanski *et al.* [15]	2024	Full text	01.2010-07.2022	retro	EO

* 3 patients reported, period of data collection not specified

** reported 4 cases, only patient case No. 2 considered as matching eligibility criteria (intended diagnosis and treatment), period of data collection not specified

*** 3 patients reported, data from period 08.2016-03.2019, nk.2017-06.2019, 05.2016-12.2019, respectively

**** 2 cohorts reported, only one cohort (stage 2 patients) considered as matching intended long-term treprostinil treatment

***** 2 patients reported, period of data collection not specified

nk = not known/not specified; R= retrospective, observational cohort studies, CR = case report, P, OL = prospective open-label studies, EO= efficacy overview, MA= meta-analysis, SO=safety overview

2. Statistical method for Bayesian meta-analysis

A Bayesian meta-analysis of the relevant existing literature in key endpoints in paediatric progressive pulmonary arterial hypertension (PAH), i.e., 6-minute walking distance (6MWD), pulmonary vascular resistance (PVR)/pulmonary vascular resistance index (PVRi), mean pulmonary arterial pressure (mPAP), Tricuspid annular plane systolic excursion (TAPSE) and World Health Organisation functional class (WHO FC) was performed.

After a careful and extensive review of the available literature on the use of treprostinil for the intended indication, we summarized the statistical information in each paper that contains relevant and interpretable information on these variables.

The statistician used Bayesian statistical methods to combine all distributions into a single approximating posterior distribution for each parameter, the meta-analytic-predictive (MAP) distribution. This in turn permits formal statements regarding the probability that treprostinil offers a clinically significant benefit in each area.

2.1. Continuous variable analysis

For the four continuous variables (6MWD, PVR, mPAP and TAPSE), the goal was to obtain a MAP distribution for the change in the variable since baseline, Δ. Since typically means and standard deviations (SDs) are reported, we approximated the Δ for each variable by a normal distribution. As all included publications were single group studies, within group mean difference μi and corresponding pooled variance Varpooled were calculated from pre- and post-treatment means and standard deviations, respectively. With the assumptions of inhomogeneity of variances and approximate independence of pre and post samples, the pooled variance of study i was calculated using the following formula:

$$\mathrm{Var}_{i\ pooled} = \frac{SD_{i\ Pre}^{2}}{n_{i\ Pre}} + \frac{SD_{i\ Post}^{2}}{n_{i\ Post}}$$

The resulting distributions were then combined into a single approximating normal MAP distribution by adding their precisions (reciprocal variances) and computing the weighted average of their means based on the post-treatment sample size.[34]

That is,

$$\Delta \sim N\left(\frac{\sum_i n_{i\,Post}\ \mu_i}{\sum_i n_{i\,Post}}, \left[\sum_i P_i\right]^{-1}\right),$$

where $n_{i\ Post}$ is the sample size of the post treatment observation, μ_i is the mean difference between post- and pre-treatment, and P_i is the precision of study i for i = 1, …, k. calculated as $1/Var_{i\ pooled}$ Using the calculated parameters and quantiles of the normal distribution 95% credible intervals can be directly calculated.

Additionally, to determine clinical significance, we computed the probability that 6MWD increased by at least 50 m, the probability that PVR decreased by at least 3 WU, mPAP decreases by at least 5 mmHg and TAPSE increases by at least 2 mm. The choice of clinically significant difference in 6MWD is based on three studies which reported estimates calculated by using different statistical methods of 33 m (range 25.1 – 38.6 m) [35], 40.7 m (range 18.7 – 74.15) [36] and 41.8 m [37]. The cut-off values for improvement in PVR and mPAP of 3 WU and 5 mmHg and 2mm for TAPSE are not based on any guidance but were assumed to be of clinical relevance for the patient in terms of PAH symptom improvement.

Alternatively, a noninformative Bayesian (i.e., likelihood-based) solution would use the study-specific precisions in the weighted average of the MAP distribution means. That is,

$$\Delta \sim N\left(\frac{\sum_i P_i\ \mu_i}{\sum_i P_i}, \left[\sum_i P_i\right]^{-1}\right).$$

Notice that in both of these expressions, all precision comes from past data; the “initial prior” on Δ is flat, so only the literature studies contribute any information to the MAP estimate. This approach is methodologically akin to the most widely employed meta-analysis assuming a common effect (CE), and therefore both produce identical point and interval estimates.[38] Using the corresponding quantiles of the normal distribution, 95% credible intervals can be calculated

from MAP distributions. These 95% credible intervals are identical with “likelihood-based” 95% confidence intervals when non-informative priors are used. For normally distributed random variables inverse-variance weighted averages can also be derived as the **maximum likelihood estimate** for the true value.

The only significant limitation of the CE model is its basic assumption that all included studies estimate the same effect. For some studies, study heterogeneity cannot be reliably estimated. More generally, in the case of only 2 available studies, the CE model might be used as a default unless there are strong arguments against the CE assumption. In the presence of between-study heterogeneity, especially with unbalanced study sizes, caution is needed in applying meta-analytical methods to few studies, as either coverage probabilities of intervals may be compromised, or they may be inconclusively wide. Bayesian estimation with a sensibly chosen prior for the between-study heterogeneity may offer a promising compromise.[39]

Nevertheless, we reported the p-values and confidence intervals of heterogeneity statistic I^2, since the point estimate of the I^2 can be biased.[40]

2.1.1. Prior robustness check

As a robustness check of the Bayesian method with precision weighting, we added a sceptical prior,

$$\Delta \sim N\left(0\,,\left[\frac{1}{10}\sum_i P_i\right]^{-1}\right).$$

That is, the prior adds the same information as an imaginary extra study having precision equal to 1/10 of the total precision in all our remaining studies, and mean 0 (i.e., the treatment had no effect). Writing $\widehat{\Delta}$ and σ_Δ^2 as the MAP mean and variance under the flat initial prior, the MAP distribution under the sceptical initial prior becomes

$$\Delta \sim N\left(\tfrac{10}{11}\,\widehat{\Delta}\,,\tfrac{10}{11}\sigma_\Delta^2\right).$$

Analogously, for the Bayesian method using sample size weighting, we assumed the sceptical prior would have variance $\frac{10}{11}\sigma_{\Delta}^{2}$ and a mean computed assuming the imaginary extra study has its zero-mean weighted by a sample size of

$$\frac{1}{10}\sum_{i} n_{i\,Post}$$

This permits utilization of the same prior robustness check with both our sample size and precision weighting approaches.

2.2. Discrete variable analysis

To analyse the variable WHO FC, we handled the categorical nature of the parameter by binarizing it, grouping together responses in classes I and II (at most mild symptoms and functional limitations) and responses in classes III and IV (marked or severe limitations). A standard meta-analytic way for combining success-failure data is the beta-binomial approach. Let θ_B and θ_A be the probabilities of being in the first, less severe category before (at baseline) and after treprostinil treatment, respectively, and let x_{iB} and n_{iB} be the number of less severe subjects and the total number of subjects reported in study i (i = 4 for WHO FC) at baseline. Then under a noninformative (uniform) prior distribution on θ_B, the posterior distribution for this parameter emerges as a beta distribution [34]

$$\theta_B \sim Beta\left(\sum_{i} x_{iB} + 1\,, \sum_{i}(n_{iB} - x_{iB}) + 1\right),$$

with a similar expression holding for θ_A as a function of the after-treatment data x_{iA} and n_{iA}. As before in the continuous case, these two beta distributions permit probabilistic statements about $\delta = \theta_A - \theta_B$, the improvement in the proportion of less severe patients due to treprostinil. In particular, obtained Bayesian point and 95% credible interval estimates for δ indicate the statistical significance of this change.

Furthermore, it must be noted that the above-mentioned analyses ignore any before-after correlation at patient level, which cannot be estimated from published summaries since they typically do not report individual patient-level data. However, were these (small positive) correlations incorporated, the effect would be to further *reduce* the estimated standard errors, making each of findings even more significant. Therefore, the results below can be thought of as *lower bounds* on the efficacy of treprostinil in the paediatric PAH population.

In the meta-analysis, it was assumed that the included publications are equally valid and mutually independent.

In order to demonstrate robustness of the estimated effect sizes, all analyses were repeated using different priors. In the case of the continuous endpoints, this involved replacing the flat prior of the primary analysis with the sceptical prior above. The result of this prior is always to attenuate the results back towards the null; it can then be checked if the results retain their clinical and statistical significance. In the case of the binary endpoint (WHO FC), the sceptical prior would correspond to a Beta (6,6) distribution. This corresponds to a thought experiment started with the flat prior, and then 10 patients of an imaginary study are added, 5 successes and 5 failures, before adding in all the other studies. This would represent a null finding in a study as large as 10% of data used in primary analysis which is comparable to the sceptical prior for continuous variables having 10% of total precision. We also included the results of an alternative noninformative prior, the Jeffreys prior, which in this case is a Beta (1/2, 1/2) distribution.

The common effect in WHO FC was calculated based on risk differences between pre and post treatment observation of proportions of patients in the I/II group. This was calculated using the R package meta.

2.2.1. Dataset inclusion sensitivity analysis

In addition to the extensive prior robustness investigation above, we also performed further sensitivity analyses that, instead of altering the prior distribution excluded specific datasets based on their contributions to the MAP distribution; namely the one most supportive of a treatment effect, or the one least supportive of this effect.

Exploratory analyses accounting for other factors of relevance for outcome (e.g. baseline WHO FC, concomitant PAH specific therapy or antiplatelet or antithrombotic therapy) and for different forms of group 1 (PAH) were planned, but due to limited availability of individual patient data, such exploratory analyses were not possible.

3. Results

3.1. Meta-analysis

3.1.1. TAPSE

Only two of the selected publications for the meta-analysis evaluated change in TAPSE resulting in limited amount of the input data precluding best- and worst-case sensitivity analysis thus informative value of the meta-analysis for this parameter is considerably reduced. For this reason, we publish results in here in the supplement material rather than in the main manuscript text. Despite of only limited data available we still conducted a primary analysis comparable to the other parameters revealing a probability of TAPSE improvement of 2 mm due to treprostinil therapy of 74% being statistically significant. Inclusion bias check was performed instead of best/worst case analysis swapping 6-12 months data for 1-3 months data in Hopper, Wang, DeMatteo *et.al* [17] and lead to a drop in probability of TAPSE improvement to 40% being still statistically significant. Nevertheless, given wide range between these two probability values, the analysis results are rather inconclusive what is likely due to limited input data.

TABLE S2: Results of meta-analysis for TAPSE

Endpoint	Type of analysis	Selected Publications	Prior*	Statistical Method	MAP Mean (SD)	$CrI_{95\%}$	Exceedance probability**	$CI_{95\%}$ I^2 [%]***	p-value****
TAPSE	Primary	Hopper 2018 6-12-m [17] Levy 2018 [16]	Flat	Sample Size	2.41 (0.63)	(1.18, 3.64)	0.744	-	-
				Precision	2.40 (0.63)	(1.17, 3.63)	0.739	n.c.	0.00013
	Inclusion bias	With Hopper at 1-3 instead of 6-12m [17]	Flat	Sample Size	1.84 (0.62)	(0.63, 3.05)	0.398	-	-
				Precision	1.84 (0.62)	(0.63, 3.06)	0.400	n.c.	0.0028
	Prior Robustness	Hopper 2018 6-12-m [17] Levy 2018 [16]	Sceptical	Sample Size	2.19 (0.60)	(1.01, 3.37)	0.624	-	-
				Precision	2.18 (0.60)	(1.00, 3.36)	0.618	-	-

*Initial priors for primary, the worst, best case and inclusion bias sensitivity analysis are flat meaning that only the literature studies contribute any information to the meta-analytic-predictive (MAP) estimate. Sceptical initial prior was added for prior robustness check. The sceptical prior adds the same information as an imaginary extra study having precision equal to 1/10 of the total precision in all our remaining studies, and mean 0 (i.e., the treatment had no effect) and zero-mean weighted by a sample size.

** Probability of clinically significant improvement on treprostinil treatment, clinical significance was defined as increase of TAPSE by ≥ 2mm

*** If n.c. – confidence interval of I2 is not calculable, because the Cochrane Q statistic is either greater than number of studies k and the number of studies k < 2 OR Cohrane Q <= k and k <= 2 (see also R library meta)

**** a noninformative Bayesian (i.e., likelihood-based) solution was used for calculation of p-values

3.2. Safety Overview

Based on treprostinil related adverse drugs reactions (ADRs) reported in the publications yielded by the literature search (as shown in **Figure S1** and **TABLE S1**) we calculated frequency of ADRs in total population of 200 paediatric patients included in these publications and compared it to frequency in adult population. For safety data in the adults we considered the long-term observations with large datasets and consistent reporting i.e. Barst, Galie *et al*. [41], who provided data for 860 patients on SC treprostinil in their long-term extension study including the patients of the two pivotal phase III studies and eight studies investigating IV administration [42-49].

Overview of systemic and administration route (i.e. SC or IV) related adverse drug reaction in both population is provided in **TABLE S3** and **TABLE S4**, respectively.

3.2.1. Systemic adverse drug reactions

TABLE S3: Safety Overview of reported systemic adverse drug reactions

**Adverse Drug Reaction Preferred Term***	**System organ class**	**Reported frequency paediatric n (%) Total (n=200)**	**Reported frequency adults n (%) Total (n=1055)****
Flushing [a)]	Vascular disorders	**31 (16)**	34 (3)
Diarrhoea [b)]	Gastrointestinal disorders	27 (14)	422 (40)
Swelling/Oedema [c)]	Vascular disorders	**27 (14)**	12 (1)
Headache/Dizziness [d)]	Nervous system disorders	26 (13)	323 (31)
Joint/muscle pain/other pain (i.e. pain in extremities) [e)]	Musculoskeletal and connective tissue disorders	24 (12)	238 (23)
Rash [f)]	Skin and subcutaneous tissue disorders	**20 (10)**	91 (9)
Itching [g)]	Skin and subcutaneous tissue disorders	**19 (10)**	0
Nausea [h)]	Gastrointestinal disorders	13 (7)	280 (27)
Jaw Pain [i)]	Musculoskeletal and connective tissue disorders	6 (3)	252 (24)
Vomiting [j)]	Gastrointestinal disorders	6 (3)	41 (4)
Fever [k)]	General disorders and administration site conditions	**4 (2)**	9 (<1)
Systemic hypotension [l)]	Vascular disorders	**2 (1)**	9 (<1)

Adverse Drug Reaction Preferred Term*	System organ class	Reported frequency paediatric n (%) Total (n=200)	Reported frequency adults n (%) Total (n=1055)**
Insomnia [m)]	Nervous system disorders	**2 (1)**	4 (<1)
Thrombocytopenia [n)]	Blood and lymphic disorders	1 (<1)	6 (<1)
Decreased appetite [o)]	Gastrointestinal disorders	**1 (<1)**	0
Tachycardia/Palpitations [p)]	Vascular disorders	1 (<1)	15 (1)
Vasodilatation	Vascular disorders	-	115 (11)
Anorexia	Gastrointestinal disorders	-	89 (8)
Dyspnoea	Respiratory, thoracic and mediastinal disorders	-	20 (2)
Loose stools	Gastrointestinal disorders	-	11 (1)
Fatigue	General disorders and administration site conditions	-	11 (1)
Cough	Respiratory, thoracic and mediastinal disorders	-	8 (<1)
Heart failure	Cardiac disorder	-	5 (<1)
Chest pain	Cardiac disorder	-	3 (<1)
Non-cardiac chest pain	General disorders and administration site conditions	-	2 (<1)
Pneumonia	Respiratory, thoracic and mediastinal disorders	-	1 (<1)
Syncope	Nervous system disorders	-	1 (<1)

*reported as one preferred term (PT) per patient
**reported frequency for adult population is based on Barst *et al.*, 2006 [41] providing data for 860 patients treated with subcutaneous (SC) treprostinil and 8 studies for in total 195 patients treated with intravenous (IV) treprostinil Benza *et al.*, 2013 [42]; Gomberg-Maitland *et al.*, 2005 [43]; Tapson *et al.*,, 2006a [44]; Tapson *et al.*, 2006b [45]; Minai *et al.*, 2013 [46]; Sitbon *et al.*, 2007 [47]; Hiremath *et al.*, 2010 [48]; El-Kersh *et al.*, 2014 [49]
a) Nelson *et al.*, 2023 [32]
b) 11, 15 and 1 cases reported in Hall *et al.*, 2019 [19], Nelson *et al.*, 2023 [32], and Serrano *et al.*, 2019 [2]; respectively
c) Nelson *et al.*, 2023 [32]
d) 14, 11 and 1 cases reported in Hall *et al.*, 2019 [19], Nelson *et al.*, 2023 [32], and Serrano *et al.,* 2019 [2]; respectively
e) 20 cases of joint/muscle pain reported in Nelson *et al.*, 2023 [32]; 1 and 3 cases of other pain reported in Levy *et al.*, 2018 [16] and Hall *et al.,* 2019 [19], respectively
f) 4 and 16 cases reported in Hall *et al.*, 2019 [19] and Nelson *et al.*, 2023 [32]; respectively
g) Nelson *et al.*, 2023 [32]
h) 12 and 1 cases reported in Hall *et al.,* 2019 [19] and Serrano et al., 2019 [32]; respectively
i) Hall *et al.*, 2019 [19]
j) 5 and 1 cases reported in Hall *et al.*, 2019 [19] and Nelson *et al.,* 2023 [32]; respectively
k) Nelson *et al.,* 2023 [32]
l) 1 and 1 case reported in Serrano *et al.*, 2019 [2] and Nelson *et al.*, 2023 [32]; respectively
m) Nelson *et al.*, 2023 [32]
n) Ablonczy *et al.*, 2018 [18]
o) Nelson *et al.*, 2023 [32]
p) Serrano *et al.,* 2019 [2]

3.2.2. Adverse drug reactions related to application route

TABLE S4: Safety overview of reported reactions related to application route

**Adverse Drug Reaction Preferred Term***	**Reported frequency paediatric n (%)**		**Reported frequency adults n (%)****	
	**SC treprostinil (n=152)*****	**IV treprostinil (n=54)*****	**SC treprostinil (n =860)**	**IV treprostinil (n =195)**
Infusion site pain [a)]	122 (80)	-	799 (93)	5 (3)
Local site infection [b)]	13 (9)	-	44 (5)	2 (1)
Infusion site reaction [c)]	5 (3)	-	700 (81)	0 (0)
Central-line infection/blood stream infection [d)]	-	14 (26)	0 (0)	8 (4)
Dislocation of implanted catheter [e)]	na	1 (2)	na	nk
Haematoma of pump pocket [f)]	na	2 (4)	na	nk
Pneumothorax [g)]	na	1 (2)	na	nk
Infusion site bleed/bruise	-	-	170 (20)	0 (0)
Hematoma	-	-	0 (0)	1 (<1)
Bacteriemia	-	-	0 (0)	1 (<1)

*reported as one preferred term (PT) per patient
** reported frequency for adult population is based on Barst *et al.*, 2006 [41] providing data for 860 patients treated with subcutaneous (SC) treprostinil and 8 studies for in total 195 patients treated with intravenous (IV) treprostinil Benza *et al.*, 2013 [42]; Gomberg-Maitland *et al.*, 2005 [43]; Tapson *et al.*,, 2006a [44]; Tapson *et al.*, 2006b [45]; Minai *et al.*, 2013 [46]; Sitbon *et al.*, 2007 [47]; Hiremath *et al.*, 2010 [48]; El-Kersh *et al.*, 2014 [49]
*** 3 patients reported in Alvarez-Fuente *et al.,* 2020 were pretreated with SC treprostinil and switched to IV administration by infusion pump system, these patients are counted in both populations (SC and IV). In addition, 3 patients in Colgazier *et al.,* 2021 received firstly SC treprostinil and were subsequently switched to IV treprostinil, these patients are counted in in both populations (SC and IV).

na=not applicable, nk= not known (data not available in the selected literature)

a) reported cases in Author, year [reference] (number of patients experiencing ADR): Ablonczy *et al.*, 2018 [18] (8), Vernetta *et al.*, 2017 [6] (2), Levy *et al.*, 2018 [16] (44), Hall *et al.*, 2019 [19] (21), Alvarez-Fuente *et al.*, 2020 [23] (1); Ono *et al.*, 2020 [22] (1), Colgazier et al., 2021 [27] (3), Nelson *et al.*, 2023 [32] (41), Faircloth *et al.*, 2023 [33] (1)
b) 12, 1, cases reported in Levy *et al.*, 2018 [16]; Alvarez-Fuente *et al.*, 2020 [23]; respectively
c) 4, 1 cases reported in Vernetta *et al.*, 2017 [6], Alvarez-Fuente *et al.*, 2020 [23]; respectively
d) reported cases in Author, year [reference] (number of patients experiencing ADR): Ivy *et al.*, 2007 [7] (7), Rowan *et al.,* 2008 [8] (4), Colgazier et al., 2021 [27] (2), Faircloth *et al.,*2023 [33] (1)
e) Desole et al., 2019 [21]
f) 1 and 1 case reported in Desole *et al.,* 2019 [21] and Álvarez-Fuente *et al.*, 2020 [23], respectively
g) Álvarez-Fuente *et al.,* 2020 [23]

4. References

1. Bajolle F, Levy M, Szezepanski I, et al. Subcutaneous Treprostinil In Pediatric Pulmonary Arterial Hypertension. B58 TREATMENT OF PULMONARY ARTERIAL HYPERTENSION: American Thoracic Society; 2010. p. A3343-A.
2. Serrano RM, Subbarao GC, Mangus RS, et al. Combination therapy for severe portopulmonary hypertension in a child allows for liver transplantation. Pediatr Transplant. 2019;23(5):e13461. Epub 2019/05/08. doi: 10.1111/petr.13461. PubMed PMID: 31062925.
3. Tella JB, Kulik TJ, McSweeney JE, et al. Prostanoids in pediatric pulmonary hypertension: clinical response, time-to-effect, and dose-response. Pulm Circ. 2020;10(4):2045894020944858. Epub 2020/12/22. doi: 10.1177/2045894020944858. PubMed PMID: 33343879; PubMed Central PMCID: PMCPMC7727065.
4. Haarman MG, Douwes JM, Ploegstra MJ, et al. The Clinical Value of Proposed Risk Stratification Tools in Pediatric Pulmonary Arterial Hypertension. Am J Respir Crit Care Med. 2019;200(10):1312-5. Epub 2019/07/13. doi: 10.1164/rccm.201902-0266LE. PubMed PMID: 31298925.
5. Miles KG, Critser PJ, Evers PD, et al. Factors leading to supranormal cardiac index in pediatric pulmonary hypertension patients treated with parenteral prostanoid therapy. Pulm Circ. 2023;13(3):e12264. Epub 20230705. doi: 10.1002/pul2.12264. PubMed PMID: 37427091; PubMed Central PMCID: PMCPMC10323166.
6. Vernetta AT, Amigo SR, Serrano II, et al. Experience with subcutaneous treprostinil in children with pulmonary arterial hypertension. Eur Respiratory Soc; 2017.
7. Ivy DD, Claussen L, Doran A. Transition of stable pediatric patients with pulmonary arterial hypertension from intravenous epoprostenol to intravenous treprostinil. The American journal of cardiology. 2007;99(5):696-8.
8. Rowan C, Barst R, Rosenzweig E. Long-Term IV Treprostinil in Pediatric Pulmonary Arterial Hypertension. American journal of respiratory and critical care medicine. 2008;177(Abstracts Issue):A696.
9. McSweeney J, Colglazier E, Becerra J, et al. Failure to tolerate continuous subcutaneous treprostinil in pediatric pulmonary hypertension patients. Pulmonary Circulation. 2023;13(2). doi: 10.1002/pul2.12224.
10. Smadja DM, Mauge L, Gaussem P, et al. Treprostinil increases the number and angiogenic potential of endothelial progenitor cells in children with pulmonary hypertension. Angiogenesis. 2011;14(1):17-27. Epub 2010/11/05. doi: 10.1007/s10456-010-9192-y. PubMed PMID: 21049284; PubMed Central PMCID: PMCPMC3040815.
11. Hollander SA, Ogawa MT, Hopper RK, et al. Treprostinil improves hemodynamics and symptoms in children with mild pulmonary hypertension awaiting heart transplantation. Pediatric transplantation. 2020;24(5):e13742.
12. Sullivan RT, Handler SS, Feinstein JA, et al. Subcutaneous Treprostinil Improves Surgical Candidacy for Next Stage Palliation in Single Ventricle Patients With High-Risk Hemodynamics. Semin Thorac Cardiovasc Surg. 2023;35(4):733-43. Epub 20220802. doi: 10.1053/j.semtcvs.2022.07.011. PubMed PMID: 35931345.
13. Levy M, Bonnet D, Mauge L, et al. Circulating endothelial cells in refractory pulmonary hypertension in children: markers of treatment efficacy and clinical worsening. PLoS One. 2013;8(6):e65114. Epub 2013/06/14. doi: 10.1371/journal.pone.0065114. PubMed PMID: 23762293; PubMed Central PMCID: PMCPMC3677895.
14. Siehr SL, Ivy DD, Miller-Reed K, et al. Children with pulmonary arterial hypertension and prostanoid therapy: long-term hemodynamics. J Heart Lung Transplant. 2013;32(5):546-52. Epub

2013/03/05. doi: 10.1016/j.healun.2013.01.1055. PubMed PMID: 23453572; PubMed Central PMCID: PMCPMC3760159.
15. Kochanski JJ, Feinstein JA, Ogawa M, et al. Younger age at initiation of subcutaneous treprostinil is associated with better response in pediatric Group 1 pulmonary arterial hypertension. Pulmonary Circulation. 2024;14(1). doi: 10.1002/pul2.12328.
16. Levy M, Del Cerro MJ, Nadaud S, et al. Safety, efficacy and Management of subcutaneous treprostinil infusions in the treatment of severe pediatric pulmonary hypertension. Int J Cardiol. 2018;264:153-7. Epub 2018/04/14. doi: 10.1016/j.ijcard.2018.03.067. PubMed PMID: 29650343.
17. Hopper RK, Wang Y, DeMatteo V, et al. Right ventricular function mirrors clinical improvement with use of prostacyclin analogues in pediatric pulmonary hypertension. Pulm Circ. 2018;8(2):2045894018759247. Epub 2018/02/27. doi: 10.1177/2045894018759247. PubMed PMID: 29480089; PubMed Central PMCID: PMCPMC5843105.
18. Ablonczy L, Tordas D, Kis E, et al. Use of subcutaneous treprostinil in pediatric pulmonary arterial hypertension—Bridge-to-transplant or long-term treatment? Pediatric transplantation. 2018;22(2):e13106.
19. Hall K, Ogawa M, Sakarovitch C, et al. Subcutaneous and intravenous treprostinil pharmacokinetics in children with pulmonary vascular disease. Journal of cardiovascular pharmacology. 2019;73(6):383-93.
20. Bacha NC, Levy M, Guerin CL, et al. Treprostinil treatment decreases circulating platelet microvesicles and their procoagulant activity in pediatric pulmonary hypertension. Pediatr Pulmonol. 2019;54(1):66-72. Epub 2018/11/30. doi: 10.1002/ppul.24190. PubMed PMID: 30485728.
21. Desole S, Richter MJ, Heine A, et al. Intravenous treprostinil via an implantable pump in pediatric pulmonary arterial hypertension. Pulm Circ. 2019;9(1):2045894018788846. Epub 2018/06/27. doi: 10.1177/2045894018788846. PubMed PMID: 29944075; PubMed Central PMCID: PMCPMC6295941.
22. Ono H, Yotani N, Kato H. Using the upper buttocks as a subcutaneous site for treprostinil infusion in children with pulmonary artery hypertension. Cardiol Young. 2020;30(7):1024-5. Epub 2020/05/27. doi: 10.1017/S1047951120001237. PubMed PMID: 32452334.
23. Alvarez-Fuente M, Garrido-Lestache E, Rivero N, et al. Implantable LENUS pro pump for treprostinil infusion in three pediatric patients. Pediatr Pulmonol. 2020;55(5):1254-8. Epub 2020/03/10. doi: 10.1002/ppul.24707. PubMed PMID: 32149476.
24. Rothman A, Cruz G, Evans WN, et al. Hemodynamic and clinical effects of selexipag in children with pulmonary hypertension. Pulm Circ. 2020;10(1):2045894019876545. Epub 20200217. doi: 10.1177/2045894019876545. PubMed PMID: 32110381; PubMed Central PMCID: PMCPMC7026823.
25. Haarman MG, Lévy M, Roofthooft MT, et al. Upfront triple combination therapy in severe paediatric pulmonary arterial hypertension. European Respiratory Journal. 2021;57(1).
26. Jackson EO, Brown A, McSweeney J, et al. Pediatric subcutaneous treprostinil site maintenance and pain control strategies from the Pediatric Pulmonary Hypertension Network. Pulmonary Circulation. 2021;11(1):204589402199445. doi: 10.1177/2045894021994450.
27. Colglazier E, Ng AJ, Parker C, et al. Safety and Tolerability of a Rapid Transition From Intravenous Treprostinil to Oral Selexipag in Three Adolescent Patients With Pulmonary Arterial Hypertension. The Journal of Pediatric Pharmacology and Therapeutics. 2021;26(5):512-6. doi: 10.5863/1551-6776-26.5.512.
28. Douwes JM, Zijlstra W. MH, Rosenzweig E. B.,Haarman M. G., Ivy D.D., Berger, R MF. Parenteral Prostanoids in Pediatric Pulmonary Arterial Hypertension: Start Early, Dose High,

Combine. 2021.
29. Handler SS, Jin J, Ogawa MT, et al. Abnormal platelet aggregation in pediatric pulmonary hypertension. Pulm Circ. 2022;12(3):e12104. Epub 20220701. doi: 10.1002/pul2.12104. PubMed PMID: 35864911; PubMed Central PMCID: PMCPMC9294293.

30. Domingo LT, Ivy DD, Abman SH, et al. Novel use of riociguat in infants with severe pulmonary arterial hypertension unable to wean from inhaled nitric oxide. Front Pediatr. 2022;10:1014922. Epub 20221201. doi: 10.3389/fped.2022.1014922. PubMed PMID: 36533232; PubMed Central PMCID: PMCPMC9751701.
31. Kavgaci A, Kula S, Incedere F, et al. Subcutaneous use of treprostinil in pediatric pulmonary hypertension patients: A report of three cases. Turk Gogus Kalp Damar Cerrahisi Derg. 2023;31(1):145-8. Epub 20230130. doi: 10.5606/tgkdc.dergisi.2023.23748. PubMed PMID: 36926146; PubMed Central PMCID: PMCPMC10012979.
32. Nelson EJ, Cook E, Nelson S, et al. Quantifying side effects and caregiver burdens of pediatric pulmonary hypertension therapies. BMC Pediatr. 2023;23(1):43. Epub 20230125. doi: 10.1186/s12887-023-03860-2. PubMed PMID: 36698086; PubMed Central PMCID: PMCPMC9875396.
33. Faircloth JM, Bhatt ND, Chartan CA, et al. Case Report: Selexipag in pediatric pulmonary hypertension: Initiation, transition, and titration. Front Pediatr. 2023;11:1050508. Epub 20230308. doi: 10.3389/fped.2023.1050508. PubMed PMID: 36969286; PubMed Central PMCID: PMCPMC10030494.
34. Carlin BP, Louis TA. Bayesian Methods for Data Analysis: Approaches for statistical inference; CHAPTER 2. The Bayes approach; CHAPTER 3. Bayesian computation; CHAPTER 4. Model criticism and selection; CHAPTER 5. The empirical Bayes approach; CHAPTER 6. Bayesian design; CHAPTER 7. Special methods and models; CHAPTER 8. Case studies: CRC Press; 2009.
35. Mathai SC, Puhan MA, Lam D, et al. The minimal important difference in the 6-minute walk test for patients with pulmonary arterial hypertension. American journal of respiratory and critical care medicine. 2012;186(5):428-33.
36. Gilbert C, Brown MC, Cappelleri JC, et al. Estimating a minimally important difference in pulmonary arterial hypertension following treatment with sildenafil. Chest. 2009;135(1):137-42.
37. Gabler NB, French B, Strom BL, et al. Validation of 6-minute walk distance as a surrogate end point in pulmonary arterial hypertension trials. Circulation. 2012;126(3):349-56.
38. Bender R, Friede T, Koch A, et al. Methods for evidence synthesis in the case of very few studies. Res Synth Methods. 2018;9(3):382-92. Epub 2018/03/06. doi: 10.1002/jrsm.1297. PubMed PMID: 29504289; PubMed Central PMCID: PMCPMC6175308.
39. Seide SE, Rover C, Friede T. Likelihood-based random-effects meta-analysis with few studies: empirical and simulation studies. BMC Med Res Methodol. 2019;19(1):16. Epub 2019/01/13. doi: 10.1186/s12874-018-0618-3. PubMed PMID: 30634920; PubMed Central PMCID: PMCPMC6330405.
40. von Hippel PT. The heterogeneity statistic I(2) can be biased in small meta-analyses. BMC Med Res Methodol. 2015;15:35. Epub 2015/04/17. doi: 10.1186/s12874-015-0024-z. PubMed PMID: 25880989; PubMed Central PMCID: PMCPMC4410499.
41. Barst RJ, Galie N, Naeije R, et al. Long-term outcome in pulmonary arterial hypertension patients treated with subcutaneous treprostinil. Eur Respir J. 2006;28(6):1195-203. Epub 2006/08/11. doi: 10.1183/09031936.06.00044406. PubMed PMID: 16899485.
42. Benza RL, Tapson VF, Gomberg-Maitland M, et al. One-year experience with intravenous treprostinil for pulmonary arterial hypertension. J Heart Lung Transplant. 2013;32(9):889-96. Epub 2013/08/21. doi: 10.1016/j.healun.2013.06.008. PubMed PMID: 23953817.
43. Gomberg-Maitland M, Tapson VF, Benza RL, et al. Transition from intravenous epoprostenol to intravenous treprostinil in pulmonary hypertension. Am J Respir Crit Care Med. 2005;172(12):1586-9. Epub 2005/09/10. doi: 10.1164/rccm.200505-766OC. PubMed PMID: 16151039.
44. Tapson VF. Safety and Efficacy of IV Treprostinil for PAH. Chest. 2006.
45. Tapson VF. Delivery of intravenous treprostinil at low infusion rates using a miniaturized infusion pump in patients with PAH. The Journal of Vascular Access. 2006.
46. Minai OA, Parambil J, Dweik RA, et al. Impact of switching from epoprostenol to IV treprostinil on treatment satisfaction and quality of life in patients with pulmonary hypertension. Respir Med. 2013;107(3):458-65. Epub 2012/12/26. doi: 10.1016/j.rmed.2012.10.023. PubMed PMID: 23266038.

47. Sitbon O, Manes A, Jais X, et al. Rapid Switch From Intravenous Epoprostenol to Intravenous Treprostinil in Patients With Pulmonary Arterial Hypertension. Journal of Cardiovascular Pharmacology. 2007;49(1):1-5. doi: 10.1097/FJC.0b013e31802b3184. PubMed PMID: 00005344-200701000-00001.
48. Hiremath J, Thanikachalam S, Parikh K, et al. Exercise improvement and plasma biomarker changes with intravenous treprostinil therapy for pulmonary arterial hypertension: a placebo-controlled trial. J Heart Lung Transplant. 2010;29(2):137-49. Epub 2009/12/22. doi: 10.1016/j.healun.2009.09.005. PubMed PMID: 20022264.
49. El-Kersh K, Ruf K, Smith JS. Rapid Inpatient Titration of Intravenous Treprostinil for Pulmonary Arterial Hypertension: Safe and Tolerable. Chest. 2014;146(4). doi: 10.1378/chest.1987360.
